# Plasma Levels of High-Mobility Group Box 1 during Peptide Vaccination in Patients with Recurrent Ovarian Cancer

**DOI:** 10.1155/2017/1423683

**Published:** 2017-04-27

**Authors:** Kayoko Waki, Kouichiro Kawano, Naotake Tsuda, Kimio Ushijima, Kyogo Itoh, Akira Yamada

**Affiliations:** ^1^Cancer Vaccine Development Division, Research Center for Innovative Cancer Therapy, Kurume University, Kurume, Japan; ^2^Department of Obstetrics and Gynecology, Kurume University School of Medicine, Kurume, Japan; ^3^Cancer Vaccine Center, Kurume University, Kurume, Japan

## Abstract

High-mobility group box 1 (HMGB1) is a nuclear protein that is known to be secreted into extracellular fluids from injured cells, activated macrophages, and tumor cells. The clinical correlation of circulating HMGB1 levels with various diseases including cancer has been reported. However, there is no information on HMGB1 levels in cancer patients treated with peptide vaccination. In the present study, we investigated the plasma levels of HMGB1 during personalized peptide vaccination in patients with recurrent ovarian cancer. Frozen plasma samples of 39 patients from previously conducted clinical trials were used in this study. HMGB1 levels were decreased after the 1st cycle of vaccination from their prevaccination levels. However, no correlation was observed between HMGB1 and overall survival (OS). The correlation between plasma HMGB1 levels and other biomarker levels was further analyzed by scatter plot, revealing that HMGB1 levels after the 1st cycle of vaccination were significantly correlated with myeloid-derived suppressor cell (MDSC) frequency after the 1st cycle of vaccination (*r* = 0.357, *p* = 0.032). Chi-square test showed that epitope spreading was significantly related with changes of HMGB1 (*p* = 0.030). These results suggest that plasma HMGB1 is a possible biomarker for cancer vaccine therapy, although direct correlation with OS has not been obtained. This trial is registered with Clinical Trial Registry under trial numbers UMIN000003083 and UMIN000001482.

## 1. Introduction

Ovarian cancer is one of the most common cancers in women worldwide. The standard first-line treatment is chemotherapy with platinum and taxane agents, and the majority of cases respond well to this treatment [1, 2]. However, most cases experienced relapse and became resistant to platinum and subsequent chemotherapeutic agents [2]. Recent studies have demonstrated that immune checkpoint blockade therapy is very effective for the treatment of melanoma and non-small-cell lung cancer, although these dramatic clinical effects were observed in only 20–30% of patients [3–5]. Promising results of immune checkpoint blockade therapy have also been reported in ovarian cancer [6].

We previously reported a clinical study of personalized peptide vaccination (PPV) for recurrent ovarian cancer patients, in which the vaccine peptides were selected from a peptide panel consisting of 31 cytotoxic T-lymphocyte- (CTL-) epitope peptides based on preexisting host immunity before vaccination [7]. In that study, we analyzed the relationship of several immune-related or inflammation-related biomarkers with the prognosis of patients and circulating lymphocyte frequency at prevaccination, and epitope spreading after the 1st cycle of vaccination was found to be significantly prognostic of overall survival (OS) [7]. However, other biomarkers were not prognostic or predictive. Therefore, the exploration of new biomarkers for cancer vaccine will be important.

High-mobility group box 1 (HMGB1) is a nuclear protein that is known to be secreted into plasma and other extracellular fluids from injured cells, activated macrophages, and tumor cells [8–11]. Circulating HMGB1 levels have been clinically and pathologically correlated with various diseases, including cancer [12]. However, there is no information on HMGB1 levels in cancer patients treated with peptide vaccination. In the present study, therefore, we investigated the plasma levels of HMGB1 during PPV in patients with recurrent ovarian cancer.

## 2. Materials and Methods

### 2.1. Plasma Samples

Frozen plasma samples that had been collected from 39 patients with recurrent ovarian cancer who were treated with PPV in previously conducted clinical trials and stored at −80°C were used in this study. The protocols of the clinical trials, including protocols for the measurement of plasma biomarkers, were approved by the Kurume University Ethics Committee and registered with the UMIN Clinical Trial Registry under trial numbers UMIN000003083 and UMIN000001482. One vaccination cycle consisted of once a week vaccination for six consecutive weeks.

Plasma HMGB1 levels were measured by using an HMGB1 ELISA kit II (Shino-Test, Sagamihara, Japan) according to the manufacturer's instructions. The detection limit was 2.5 ng/mL in our system.

### 2.2. Statistical Analysis

The statistical analyses were performed using JMP Pro version 12 software (SAS Institute, Cary, NC). Changes in the HMGB1 levels between prevaccination and postvaccination samples were analyzed by paired *t*-test. The correlation between HMGB1 levels and OS was analyzed by log-rank test. The comparisons between plasma HMGB1 levels and other biomarkers were analyzed by *t*-test and chi-square test.

## 3. Results

### 3.1. Plasma Levels of HMGB1 during Peptide Vaccination

**Figure 1:**
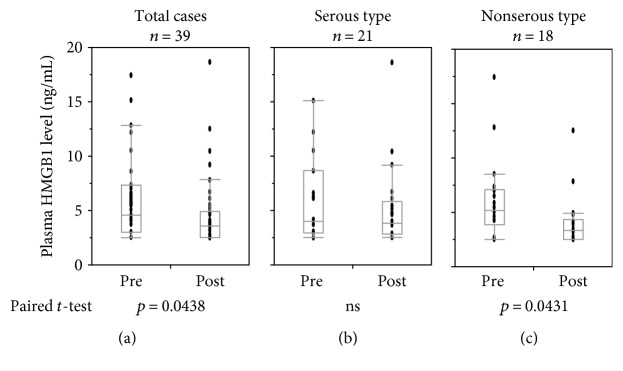
Plasma levels of HMGB1 during peptide vaccination. Plasma HMGB1 levels of pre- and post-1st cycle vaccination samples of the total 39 cases (a), the 21 serous-type cases (b), and the 18 non-serous-type cases (c) are shown. ns: Not significant.

Frozen plasma samples of 39 patients with recurrent ovarian cancer who had been treated with PPV in clinical trials were used in this study. The HMGB1 levels after the 1st cycle of vaccination (postvaccination) (4.63 ± 3.25 ng/mL) were significantly lower than the prevaccination values (5.96 ± 3.69 ng/mL) (*p* = 0.043) (Figure 1). The 39 patients consisted of patients with serous adenocarcinoma (*n* = 21), endometrioid carcinoma (*n* = 7), clear-cell carcinoma (*n* = 3), mucinous adenocarcinoma (*n* = 3), squamous cell carcinoma (*n* = 1), undifferentiated malignant tumor (*n* = 2), and others (*n* = 2). The patients were divided into two groups, that is, serous type (*n* = 21) and nonserous type (*n* = 18), for subgroup analyses. Prevaccination HMGB1 levels of the serous and nonserous types were, respectively, 5.87 ± 3.69 ng/mL and 6.07 ± 3.79 ng/mL, and those of the postvaccination group were 5.16 ± 3.75 ng/mL and 4.00 ± 2.50 ng/mL, respectively. Therefore, the significant decrease in HMGB1 levels after the 1st cycle of vaccination found in the overall group of 39 patients was confirmed in the non-serous-type group (*n* = 18, *p* = 0.043) but not in the serous type group.

### 3.2. Relationship between HMGB1 Levels and Overall Survival

**Figure 2:**
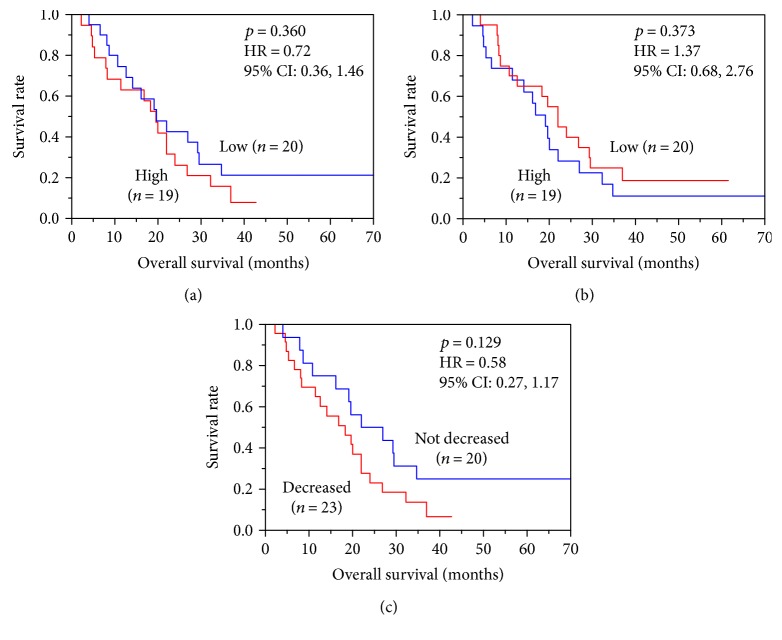
The relationship between plasma HMGB1 levels and overall survival (OS) was analyzed by Kaplan-Meier plot analysis. Patients (*n* = 39) were divided into high and low plasma HMGB1 groups based on the (a) pre- and (b) postvaccination levels, and the correlation between these subgroups and OS was analyzed. (c) Patients (*n* = 39) were also divided into decreased and not-decreased groups based on the changes of plasma HMGB1 between the prevaccination and postvaccination samples, and the relation between these subgroups and OS was plotted.

Patients were divided into high-HMGB1 group and low-HMGB1 group according to whether their pre- or postvaccination levels of plasma HMGB1 were higher or lower than the median value. Patients were also divided into a decreased or a not-decreased group according to the changes between their pre- and postvaccination values, and the correlations between these groups and OS were analyzed by Kaplan-Meier plot analysis (Figure 2). No significant correlations were found between the plasma HMGB1 levels and OS in either the pre- or postvaccination samples (Figures 2(a) and 2(b), resp.). Changes of plasma HMGB1 levels were similarly not a significant prognostic factor in OS (Figure 2(c)). It is noted that 20% of patients belonged to the low-HMGB1 prevaccination group and that 25% of the not-decreased group were long-term survivors with >37 months of OS.

### 3.3. Comparison between Plasma HMGB1 Levels and Other Biomarkers

**Figure 3:**
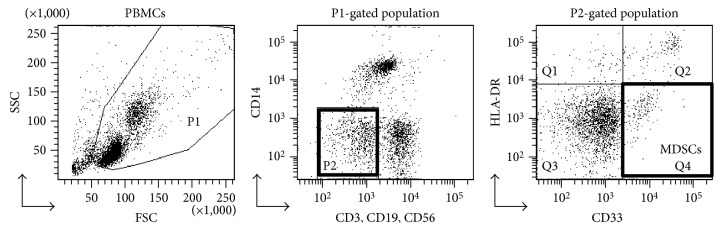
Gating strategy for the identification of MDSCs in the peripheral blood of patients. PBMCs were stained with anti-CD3-FITC, anti-CD19-FITC, anti-CD56-FITC, anti-CD33-APC, anti-HLA-DR-PE/Cy7, and anti-CD14-APC/Cy7 Abs. In the cell subset negative for the lineage markers (CD3, CD19, CD56, and CD14) and HLA-DR, MDSCs were identified as CD33^+^.

**Figure 4:**
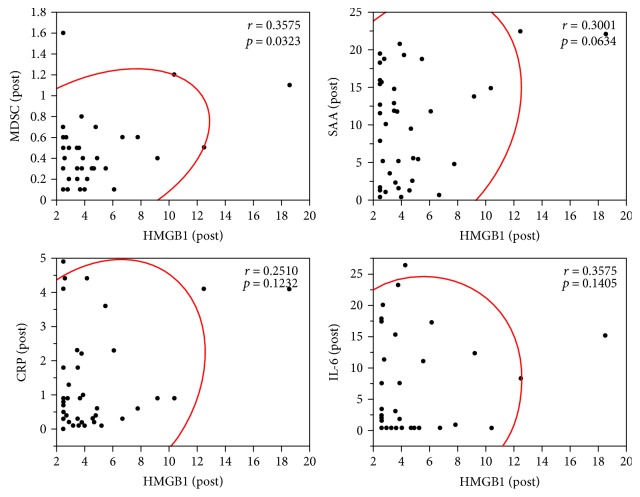
Correlations between postvaccination plasma HMGB1 levels and postvaccination levels of MDSC frequency, SAA, CRP, or IL-6 were analyzed by scatter plot.

In our previous analysis of this clinical study, we reported that epitope spreading after the 1st cycle of vaccination was a significant prognostic factor correlated to OS [7]. The other markers used in the previous study were as follows: IgG and CTL responses to the vaccinated peptides, frequency of circulating myeloid-derived suppressor cells (MDSCs), plasma levels of serum amyloid A (SAA), C-reactive protein (CRP), and interleukin-6 (IL-6). MDSCs in the peripheral blood were defined as CD33^+^ CD3^−^ CD14^−^ CD19^−^ CD56^−^ HLA-DR^−^ cells [7]. Gating strategy for the identification of MDSCs is shown in Figure 3. HMGB1 levels and previously measured data of the biomarker levels are shown in Table 1. Precise data of IgG and CTL responses and epitope spreading were previously published, and thus, these results are only summarized in Table 1. Correlations between the plasma HMGB1 levels and the other biomarker levels were analyzed by scatter plot. The postvaccination HMGB1 levels were significantly correlated with the postvaccination MDSC frequency (*r* = 0.357, *p* = 0.032) but not with the postvaccination values of SAA, CRP, or IL-6 (Figure 4). Prevaccination values or changes between the pre- and postvaccination values of HMGB1 were not correlated with the corresponding values of SAA, CRP, or IL-6 (data not shown).

Changes of plasma HMGB1 levels were categorized into three groups, an increase, a decrease, and a no-change group. Similarly, changes in each of the other biomarkers were categorized as simply “yes” or “no” (Table 2). The proportions of the categories of HMGB1 and the other biomarkers were compared by chi-square test. The results showed that epitope spreading was significantly related with changes of HMGB1 (*p* = 0.030) and that IgG and CTL responses, MDSC, SAA, CRP, and IL-6 were independent of HMGB1.

## 4. Discussion

HMGB1 is a representative member of the damage-associated molecular patterns (DAMPs), also called alarmins, which are molecules released from necrotic cells as intrinsic danger signals which induce inflammation and trigger innate immunity [8]. Toll-like receptor (TLR) 2 or 4 and receptor for advanced glycation end products (RAGE) are known to be receptors of HMGB1 [13, 14]. In addition to the role of initiator of innate immunity, HMGB1 also exerts an immunosuppressive effect through the T cell immunoglobulin domain and mucin domain 3 (TIM3), an immune checkpoint molecule on activated T cells [15]. Recently, we found that HMGB1 inhibitors, such as glycyrrhizin, exhibited an immunopotentiating effect in combination with an innate immunity receptor-related adjuvant in a murine peptide vaccine model [16]. These facts indicate that HMGB1 has two opposite functions, that is, it both initiates and suppresses immunity.

Circulating HMGB1 levels have been clinically and pathologically correlated with various diseases, including cancer [12, 17]. High levels of HMGB1 have been correlated with worsened disease outcome in most types of cancers, including colorectal cancer, bladder cancer, and hepatocellular carcinoma [12]. However, the opposite results were also obtained in some cancers—namely, high levels of HMGB1 were correlated with better prognosis in patients with esophageal cancer and gastric cancer [12]. These results were obtained from patients in various clinical settings, although none were undergoing immune therapy. In fact, to our knowledge, there has been only one report on the HMGB1 levels of patients treated with immunotherapy: Gebhardt et al. [18] reported the possibility of circulating HMGB1 as a novel predictive marker for melanoma patients who may benefit from ipilimumab (anti-CTLA-4 antibody) therapy. In the present study, therefore, we investigated the plasma levels of HMGB1 in patients with ovarian cancer treated with peptide vaccination. The presence of circulating HMGB1 in patients with ovarian cancer has been reported, and the levels were significantly higher than those in patients with benign ovarian tumors or healthy donors, and the levels in recurrent patients were higher than those in nonrecurrent patients [19, 20].

In the present study, plasma HMGB1 levels were decreased after the 1st cycle of vaccination (Figure 1). The most plausible mechanism of this decrease in HMGB1 is that the tumor mass was reduced by vaccination. However, in most of the cases studied, the best clinical response was progressive disease [7], and thus, it was hard to confirm that such a mechanism was operative. Further accumulation and analysis of biomarkers may clarify whether this mechanism plays a role. Approximately 20–25% of patients belonging to the group with low prevaccination HMGB1 or the not-decreased group were long-term survivors with >37 months of OS (Figure 2). These results may suggest that HMGB1 could be used as a predictive and/or prognostic marker of peptide vaccination. We also found that postvaccination plasma HMGB1 levels were significantly correlated with the post-MDSC frequency (Figure 3). It is known that chronic inflammation facilitates malignancy by inducing the accumulation and increasing the potency of MDSCs [21]. HMGB1 is an initiator of inflammation and has been reported to play a role in the development of MDSCs [22]. CCL2, a member of C-C chemokine family, is known as a major cytokine for the recruitment of myeloid cells including MDSCs from the bone marrow to cancer tissues [23, 24], and induction of CCL2 in various cells by HMGB1 has also been reported [25, 26]. These facts support our findings. Epitope spreading has been reported as an immune response-related prognostic marker of good outcome in our previous analysis of this clinical trial [7]. Epitope spreading was not observed in the cases with increased HMGB1 in this study. Thus, epitope spreading might be due to the suppression of immune responses by MDSCs induced by HMGB1.

## 5. Conclusion

Our present data suggest that plasma HMGB1 may be a predictive and/or prognostic marker for cancer vaccine therapy, although at the present time, there remains no direct correlation with OS. Future clinical studies on the circulating HMGB1 levels in large numbers of patients will be needed to clarify the usefulness of HMGB1 in immunotherapy.

## Figures and Tables

**Table 1:** HMGB1 and other biomarkers of patients with ovarian cancer treated with peptide vaccination^†^.

Case	Overall survival (months)	IgG response	CTL response	Epitope spreading	HMGB1^‡^ (ng/mL)			MDSC^‡^ (%)			SAA^‡^ (mg/dL)			CRP^‡^ (mg/dL)			IL-6^‡^ (pg/mL)		
					Pre	Post		Pre	Post		Pre	Post		Pre	Post		Pre	Post	
F-018	19.1	No	No	No	<2.5	2.7	NC	0.3	0.6	I	10.5	5.2	D	0.3	0.4	NC	2	11	I
FOV-003	20.0	Yes	Yes	Yes	5.9	<2.5	D	0.2	0.3	NC	1.5	19.5	I	0.0	0.9	I	0	0	NC
FOV-004	5.3	Yes	Yes	Yes	10.6	<2.5	D	0.6	0.5	NC	3.2	11.6	I	0.1	0.3	I	2	3	NC
FOV-005	11.4	Yes	Yes	Yes	5.7	<2.5	D	0.6	0.5	NC	0.4	12.7	I	0.2	0.8	I	0	2	I
FOV-006	34.7	Yes	Yes	Yes	<2.5	2.9	NC	0.5	0.5	NC	0.7	1.1	NC	0.1	0.2	I	0	0	NC
FOV-008	14.1	Yes	Yes	No	4.3	2.8	NC	0.1	0.1	NC	2.4	18.8	I	0.1	0.9	I	0	0	NC
FOV-009	8.1	Yes	Yes	Yes	4.5	3.9	NC	0.5	0.4	NC	20.4	20.8	NC	0.7	1.0	NC	0	0	NC
FOV-010	>69.7	Yes	Yes	Yes	<2.5	<2.5	NC	0.1	0.3	I	0.3	7.9	I	0.0	0.5	I	0	0	NC
FOV-012	16.1	Yes	Yes	No	<2.5	<2.5	NC	1.1	1.6	NC	6.3	1.7	D	0.1	0.5	I	0	0	NC
FOV-013	4.0	No	Yes	No	<2.5	3.7	NC	0.1	0.1	NC	11.8	11.8	NC	0.9	0.9	NC	10	23	I
FOV-014	32.2	Yes	Yes	Yes	5.0	3.5	NC	0.3	0.3	NC	12.8	14.8	NC	0.1	0.3	I	0	0	NC
FOV-015	10.7	Yes	No	No	4.0	10.4	I	1	1.2	NC	2.9	14.9	I	0.6	0.9	NC	0	0	NC
FOV-016	29.5	Yes	Yes	No	3.8	9.2	I	0.6	0.4	NC	12.1	13.8	NC	1.0	0.9	NC	6	12	I
FOV-019	>61.3	Yes	No	Yes	2.7	4.9	NC	0.6	0.4	NC	1.4	5.6	I	0.1	0.6	I	0	0	NC
FOV-022	22.0	Yes	Yes	No	4.6	3.2	NC	0.5		—^§^	1.8	3.6	I	0.4	0.1	D	0	0	NC
FOV-023	16.8	Yes	Yes	Yes	6.5	3.5	NC	0.1	0.2	I	13.8	11.9	NC	1.5	2.3	NC	2	15	I
FOV-024	8.7	No	No	No	<2.5	6.1	I	0.2	0.1	D	8.9	11.8	NC	0.8	2.3	I	0	17	I
FOV-026	>45.7	Yes	Yes	Yes	3.1	5.2	NC			NC	4.2	5.5	NC	0.1	0.1	NC	0	0	NC
FOV-027	36.9	Yes	Yes	Yes	12.8	7.8	NC	0.2	0.6	I	2.6	4.8	NC	0.0	0.6	I	0	0.5	I
FOV-028	>42.7	Yes	Yes	No	17.4	<2.5	D	0.2	0.7	I	0.3	0.4	NC	0.3	0.0	D	1.2	1.1	NC
FOV-030	22.0	Yes	Yes	Yes	8.5	12.5	NC	0.4	0.5	NC	6.1	22.5	I	0.4	4.1	I	0	8	I
FOV-031	19.6	Yes	No	Yes	4.2	4.7	NC	0.3	0.3	NC	8.2	9.5	NC	0.1	0.2	I	0	0	NC
FOV-032	12.6	Yes	Yes	Yes	4.2	3.8	NC	0.6	0.3	D	0.3	5.2	I	0.2	2.2	I	0	7.2	I
FOV-033	26.9	Yes	Yes	Yes	<2.5	3.5	NC	0.4	0.5	NC	1.6	12.9	I	0.1	1.8	I	0	2.7	I
FOV-034	26.8	Yes	No	Yes	12.2	6.7	NC	0.7	0.6	NC	0.6	0.7	NC	0.1	0.3	I	0	0	NC
FOV-036	24.0	Yes	Yes	Yes	5.4	4.2	NC	0.2	0.2	NC	1.8	19.3	I	1.9	4.4	I	0.9	26.2	I
FOV-037	4.6	No	No	No	7.3	2.6	D	0.7	0.4	NC	0.7	15.8	I	1.4	4.4	I	4.6	19.8	I
FOV-038	7.9	No	No	No	15.1	18.6	NC	1.4	1.1	NC	11.6	20.9	NC	1.7	3.6	I	4.5	14.9	I
FOV-039	>10.3	Yes	No	No	3.0	<2.5	NC			—^§^	0.9	1.3	NC	0.4	0.7	NC	0	1.6	I
FOV-040	2.2	No	No	No	6.1	<2.5	D	0.8	0.6	NC	14.1	15.5	NC	1.0	1.8	NC	3.9	7.2	NC
FOV-041	18.3	Yes	No	Yes	8.6	3.6	D	0.8	0.5	NC	2.1	2.3	NC	0.1	0.1	NC	0	0	NC
FOV-042	>37	Yes	No	No	4.0	4.0	NC	0.3	0.1	D	0.3	0.4	NC	0.1	0.1	NC	0	0	NC
FOV-043	29.2	Yes	Yes	Yes	2.9	4.6	NC	0.2	0.3	NC	13.4	1.3	D	2.0	0.3	D	12.5	0	D
FOV-044	19.6	Yes	Yes	Yes	10.4	2.9	D	0.1	0.2	I	1.0	10.1	I	0.0	1.3	I	0	0	NC
FOV-045	8.3	Yes	Yes	Yes	6.3	5.5	NC	0.2	0.3	NC	1.6	18.8	I	0.7	3.6	I	1.8	10.7	I
FOV-046	6.6	Yes	Yes	No	3.7	<2.5	NC	0.3	0.1	D	21.0	16.0	NC	3.8	4.1	NC	11.4	17.1	NC
FOV-047	>35.5	Yes	Yes	Yes	8.7	3.8	D	0.5	0.8	NC	6.3	1.6	D	0.4	0.2	D	16.3	1.4	D
FOV-048	4.7	Yes	No	No	7.0	<2.5	D	0.5	0.3	NC	21.9	18.3	NC	3.9	4.9	NC	2.1	17.6	I
FOV-049	22.0	Yes	No	Yes	6.6	4.8	NC	1	0.7	NC	4.2	2.6	NC	0.2	0.4	I	0	0	NC

^†^^‡^^§^Blood samples of patients were collected before (pre) and after (post) the 1st cycle of vaccination, and HMGB1 and other biomarkers were measured. Response criteria of IgG, CTL, and epitope spreading were previously reported; post values were compared with prevalues, and if the postvalue was ≥150% or ≤50% of prevalue, it was considered increased or decreased, respectively. I: increased; D: decreased; NC: no change; samples were not available.

**Table 2:** Comparison of HMGB1 and other biomarkers.

	IgG response		CTL response		Epitope spreading		MDSC increase		SAA increase		CRP increase		IL-6 increase	
	*n* = 39		*n* = 39		*n* = 39		*n* = 37		*n* = 39		*n* = 39		*n* = 39	
		*n*		*n*		*n*		*n*		*n*		*n*		*n*
HMGB1 increase (I)	Yes	2	Yes	1	Yes	0	Yes	0	Yes	1	Yes	1	Yes	2
	No	1	No	2	No	3	No	3	No	2	No	2	No	1
HMGB1 no change (NC)	Yes	23	Yes	18	Yes	17	Yes	4	Yes	9	Yes	16	Yes	11
	No	3	No	8	No	9	No	20	No	17	No	10	No	15
HMGB1 decrease (D)	Yes	8	Yes	6	Yes	6	Yes	2	Yes	5	Yes	5	Yes	3
	No	2	No	4	No	4	No	8	No	5	No	5	No	7
*p* value^†^														
I versus not I (NC+D)		0.3698		0.2475		0.0307^∗^		0.4267		0.8493		0.4015		0.3473
D versus not D (NC+I)		0.639		0.7538		0.939		0.7039		0.3844		0.6355		0.4111

							MDSC decrease		SAA decrease		CRP decrease		IL-6 decrease	
							*n* = 37		*n* = 39		*n* = 39		*n* = 39	
								*n*		*n*		*n*		*n*

HMGB1 increase (I)							Yes	1	Yes	0	Yes	0	Yes	0
							No	2	No	3	No	3	No	3
HMGB1 no change (NC)							Yes	3	Yes	3	Yes	2	Yes	1
							No	21	No	23	No	24	No	25
HMGB1 decrease (D)							Yes	0	Yes	1	Yes	2	Yes	1
							No	10	No	9	No	8	No	9
*p* value														
I versus not I (NC+D)								0.19		0.5422		0.5422		0.6751
D versus not D (NC+I)								0.1975		0.9753		0.2389		0.418

^†^^∗^ *p* values of chi-square test are shown; statistically significant.
